# Early clinical exposure as a highly interesting educational program for undergraduate medical students: an interventional study

**DOI:** 10.1186/s12909-023-04244-x

**Published:** 2023-05-01

**Authors:** Mina AkbariRad, Majid Khadem-Rezaiyan, Sahar Ravanshad, Mahdi Rafiee, Abdollah Firoozi, Seyed Ali Zolfaghari, Hamid Reza Aghaei, Reyhaneh Zadehahmad, Setareh Azarkar, AmirAli Moodi Ghalibaf

**Affiliations:** 1 Department of Internal Medicine, Faculty of Medicine, Mashhad University of Medical Sciences, Mashhad, Iran; 2 Department of Community Medicine, Faculty of Medicine, Mashhad University of Medical Sciences, Mashhad, Iran; 4 Student Research Committee, Faculty of Medicine, Mashhad University of Medical Sciences, Mashhad, Iran; 5 Mashhad University of Medical Sciences, Mashhad, Iran; 6 Present Address: Student Research Committee, Birjand University of Medical Sciences, Birjand, Iran

**Keywords:** Medical Education, Medical students, Curriculum, Learning, Attitude

## Abstract

**Background:**

Training professional medical experts is so much dependent on the efficacy of the medical curriculum. Bearing this in mind, we aimed to evaluate the attitude of the undergraduate medical students toward the Early clinical exposure (ECE) program as a facilitator transition to the clinical phase.

**Methods:**

This quasi-experimental study was conducted on undergraduate medical students at the Mashhad University of Medical Sciences, Mashhad, Iran who were transferring from the pre-clinical course to the externship course from 2021 to 2022 by census method (i.e. all eligible students were included and no sampling was performed). An eight-session ECE intervention was performed on the participants by two professors of the Internal medicine department of Ghaem Hospital, Mashhad University of Medical Sciences, Mashhad, Iran. The participants’ attitude toward the program and the program quality was assessed with the valid and reliable scale developed by Mirzazadeh et al. (Cronbach’s alpha = 0.72). Statistical analyses were performed by SPSS software (version.16) with a statistically significant level of less than 0.05.

**Results:**

A total of 118 undergraduate medical students were enrolled in the study. Our results revealed that this program could familiarize (n = 95,81.2%)the students with the role of basic sciences knowledge in clinical settings, and 104(88.9%) participants believed that this intervention could motivate them toward learning more. The data revealed that this program was highly interesting for international students. There was a significant differentiation between Iranian and international students in familiarity with doctoring skills in medicine(P < 0.001), familiarity with the roles and responsibilities of clinical students(P < 0.001), and utility of early clinical exposure and providing more experiences(P < 0.001). According to the students’ reports, the major strengths of the program were familiarizing themselves with the clinical fields, having excellent instructors, and performing admirable training. On the other hand, the major weakness of the program was the short duration and the high population of participants in each group.

**Conclusions:**

The ECE program had a positive impact on the students’ satisfaction with medical education, and it also enhanced their understanding of the role they will play as future physicians. Therefore, we recommend that this program be implemented as a part of the medical education curriculum in medical universities.

## Background

From the beginning of creation, human health has been considered the most important wealth of an individual. Therefore, as medicine is directly related to human health, it is also of great importance [1, 2]. Previous studies have indicated that the training of expert medical clinicians is closely dependent on their education, emphasizing the significant role of medical education in the future training of physicians [3, 4]. Based on current knowledge, separating the medical education curriculum into pre-clinical and clinical courses has been determined to have a direct and indirect impact on clinical courses, ultimately leading to the training of professional physicians [5–7]. To respond to the need for training professional expert physicians, various vertical and horizontal integrated practical interventions have been introduced to medical education curricula in different universities [8–10].

One such intervention is Early Clinical Exposure (ECE), which integrates the knowledge of basic and clinical sciences and the psychosocial aspects of medical practice to move medical education towards the real context of practice, ultimately leading to the perfect training of professional physicians [11]. This educational program facilitates medical students’ transition to the clinical phase, increases their motivation and satisfaction, and makes them more aware of the application of basic sciences in medical practice. It also helps them develop a professional identity and boosts their confidence in handling patients’ problems in practice [12, 13]. The importance of ECE is increasing every day. A study by Başak et al. demonstrated that ECE programs are running in 40 medical schools in 16 countries in Europe [14].

Despite the well-established value of ECE in medical education [15], it is defined in various terms. However, one of the most exact definitions can be stated as “*authentic human contact in a social or clinical context that enhances the learning of health, illness and/or disease, and the role of the health professional, occurring in the early or preclinical years of undergraduate education*” [14, 16]. There are still various opinions about its context to find out the best unique program. Therefore, the present study is designed to develop a new unique ECE program and investigate the attitude of undergraduate medical students toward early clinical exposure at Mashhad University of Medical Sciences, Mashhad, Iran. This study can lead to improvement in the medical education curriculum.

## Methods

### Study design and participant

The present quasi-experimental study was conducted on the undergraduate medical students at Mashhad University of Medical Sciences, Mashhad, Iran from 2021 to 2022 to investigate the role of early clinical exposure programs in medical students’ clinical overview. This study was approved by the ethics committee of Mashhad University of Medical Sciences, Mashhad, Iran (code: IR.MUMS.REC.1400.137); All participants were informed about the study procedures, and written informed consent was obtained from all of them.

All Fourth-year medical students in the first semester of the 2021–2022 academic year of Mashhad University of Medical Sciences, Mashhad, Iran entered the study by census method (i.e. all eligible students were included who were 120 medical students who have the inclusion criteria of the study). On the other hand, being unwilling to participate in the survey or missing any interventional sessions were considered participants’ exclusion criteria.

### Procedures

The interventions for all the participants were provided as training sessions by two professors of the internal medicine department of Ghaem Hospital, Mashhad University of Medical Sciences, Mashhad, Iran. The intervention was conducted during a week between the end of the pre-clinical courses and the initiation of the externship courses of the undergraduate medical students. Participants were split into twelve groups, each consisting of ten medical students. The following training sessions were provided for each group:

1. introduction to externship courses, including general characteristics, extern duties, introduction to hospital wards, studying during externship courses, utilizing available facilities, and principles of infection control in hospitals.
2. basic principles of history taking from patients.
3. basic principles of physical examination and review of systems.
4. introduction to hospital and patient legal documents, how to read patient files, and writing course history of diseases.
5. communication with patients and their companions, and empathy rules.
6. basic principles of clinical reasoning.
7. introduction to Hospital Health Data System (HIS) and picture archiving and communication system (PACS).
8. introduction to staff roles and duties in hospitals and medical centers.

During each session, the case-based learning approach was conducted in each session; in detail, the professors and students discussed each clinical case from various appropriate aspects in each session. Real clinical cases were presented step-by-step to challenge the reasoning ability of participating medical students. Additionally, basic science questions related to these cases were asked of the medical students to encourage them to apply their basic science knowledge in solving patient problems.

### Outcomes

Following the interventional sessions mentioned above, the participants’ attitudes towards early clinical exposure were assessed using a scale developed by Mirzazadeh et al. in 2016. This scale consisted of three open-ended questions and seven structured questions, rated on a five-point Likert scale (strongly agree = 5 points, agree = 4 points, neutral = 3 points, disagree = 2 points, and strongly disagree = 1 point). The questionnaire was developed in seven steps according to the Mirzazadeh et al. study. Firstly, items were generated based on a critical review of the literature and informal interviews with clinical faculty involved in teaching medical students. Secondly, the questionnaire was piloted on a sample of 20 medical students to ensure clarity. The validity (e.g. face validity, content validity, criterion validity, and construct validity) and reliability of the scale were established by Mirzazadeh et al. (Cronbach’s alpha = 0.72) [17]. Demographic data, including gender, age, nationality, history of failing courses, grade point average, and national university entrance exam rank were also collected.

#### Data Analysis

Statistical analysis was conducted using SPSS software version 16 (SPSS Inc., Chicago, USA –version 16). Descriptive statistics for the demographic characteristics were presented by the mean and standard deviation for quantitative variables and frequency (percentage) for qualitative variables in the question of the survey. The independent t-test and Pearson correlation were used to present the relationship between variables. A P-value less than 0.05 was considered as the significance level. Moreover, the three open-ended questions were assessed by three different experts to extract the main idea of each phrase. In detail, at the first step, the participating students’ responses were extracted by two experts who separately read and coded them and the third one reviewed the codes and shoveled the disagreement; Then they checked and matched these codes and merged some of them if necessary.

## Results

The present study enrolled 118 medical students (61 males and 56 females) at Mashhad University of Medical Sciences, Mashhad, Iran, meeting the study’s inclusion and exclusion criteria. The mean age of the participants was 21.79 ± 1.161 years. Among the enrolled medical students, 113 (98.3%) were Iranian, while the remaining two (1.7%) were Lebanese. The studied population had a grade point average of 17.36 ± 0.92, with only two (1.7%) having failed some courses in their academic transcript. In terms of the national university entrance exam rank, the best person had achieved the second position among national university entrance exam participants but the median of them was ranked at 702 in the exam.

### Quantitative Assessment

According to the students’ views towards early clinical exposure questions, it was revealed that this program could familiarize (81.2%) of the students with the role of basic sciences knowledge in medicine and the way to apply it in clinical settings. Not only did 87% of the participants’ state that the early clinical exposure program familiarized them with the doctoring skills in medicine but also 88.9% of the intervened medical students believe that this intervention could motivate them toward learning more. Also, 83.6% of students mentioned that group discussion during the grand round could help them to reflect on their experiences and share them with others. Furthermore, 66.7% of students agreed (completely agree/agree) with the usefulness of the grand rounds. Generally, 94.8% of the participating medical students in the ECE program stated that the current program was a good experience and contended they were eager to attend such programs more (Table 1).

**Table 1:** Students’ views towards early clinical exposure

Question	Strongly agree (%)	Agree (%)	Neutral (%)	Disagree (%)	Strongly disagree (%)
**Familiarity with the role of basic sciences knowledge in clinical settings**	45 (38.5)	50 (42.7)	16 (13.7)	5 (4.7)	1 (0.9)
**Familiarity with doctoring skills in medicine**	60 (52.2)	40 (34.8)	11 (9.6)	3 (2.6)	1 (0.9)
**Motivation toward learning more**	45 (38.5)	59 (50.4)	11 (9.4)	2 (1.7)	0 (0)
**Familiarity with the roles and responsibilities of clinical students**	62 (53.9)	47 (40.9)	6 (5.2)	0 (0)	0 (0)
**Usefulness of participation in ground rounds**	42 (35.9)	36 (30.8)	32 (27.4)	4 (3.4)	3 (2.6)
**Providing opportunities to discuss and share knowledge**	52 (44.8)	45 (38.8)	10 (8.6)	3 (2.6)	6 (5.2)
**Utility of early clinical exposure and providing more experiences**	63 (53.8)	48 (41)	6 (5.1)	0 (0)	0 (0)

The analysis of the quantitative questions about students’ views towards early clinical exposure revealed that there were no significant differences (p > 0.05) between males and females in all of the questions about students’ views towards early clinical exposure. In terms of having a history of failed some courses, there were no significant differences (p > 0.05) between those who had a history of failed some courses and who did not have, except in two questions including “Usefulness of participation in ground rounds” (p = 0.043) and “Utility of early clinical exposure and providing more experiences” (p = 0.000). Being Iranian or non-Iranian was the characteristics in which there were seen significant differences in three questions including “Familiarity with doctoring skills in medicine” (p < 0.001), “Familiarity with the roles and responsibilities of clinical students” (p = 0.000), and “Utility of early clinical exposure and providing more experiences” (p < 0.001) (Table 2).

**Table 2:** Students’ views towards early clinical exposure based on their gender, history of failed some courses, and nationality

Question	Variable		Frequency of responses	Mean ± SD	P-value*
**Familiarity with the role of basic sciences knowledge in clinical settings**	Gender	Male	60	1.22 ± 0.846	0.265
		Female	56	1.04 ± 0.894	
	History of failed some courses	Not have	107	1.13 ± 0.870	0.70
		Have	2	0.00 ± 0.00	
	Nationality	Iranian	112	1.11 ± 0.874	0.153
		Non- Iranian	2	2.00 ± 0.00	
**Familiarity with doctoring skills in medicine**	Gender	Male	58	1.41 ± 0.773	0.350
		Female	56	1.27 ± 0.884	
	History of failed some courses	Not have	106	1.35 ± 0.840	0.801
		Have	2	1.50 ± 0.707	
	Nationality	Iranian	110	1.32 ± 0.834	< 0.001
		Non- Iranian	2	2.00 ± 0.00	
**Motivation toward learning more**	Gender	Male	60	1.30 ± 0.619	0.426
		Female	56	1.20 ± 0.773	
	History of failed some courses	Not have	107	1.26 ± 0.691	0.126
		Have	2	0.50 ± 0.707	
	Nationality	Iranian	112	1.23 ± 0.697	0.124
		Non- Iranian	2	2.00 ± 0.00	
**Familiarity with the roles and responsibilities of clinical students**	Gender	Male	59	1.53 ± 0.537	0.430
		Female	55	1.44 ± 0.660	
	History of failed some courses	Not have	106	1.49 ± 0.605	0.983
		Have	2	1.50 ± 0.707	
	Nationality	Iranian	110	1.46 ± 0.601	< 0.001
		Non- Iranian	2	2.00 ± 0.00	
**Usefulness of participation in ground rounds**	Gender	Male	60	0.97 ± 1.057	0.694
		Female	56	0.89 ± 0.947	
	History of failed some courses	Not have	107	0.94 ± 0.989	0.043
		Have	2	-0.50 ± 0.707	
	Nationality	Iranian	112	0.90 ± 1.004	0.126
		Non- Iranian	2	2.00 ± 0.00	
**Providing opportunities to discuss and share knowledge**	Gender	Male	59	1.10 ± 1.045	0.629
		Female	56	1.20 ± 1.052	
	History of failed some courses	Not have	107	1.18 ± 1.017	0.657
		Have	2	1.50 ± 0.707	
	Nationality	Iranian	111	1.13 ± 1.054	0.246
		Non- Iranian	2	2.00 ± 0.00	
**Utility of early clinical exposure and providing more experiences**	Gender	Male	60	1.48 ± 0.537	0.991
		Female	56	1.48 ± 0.660	
	History of failed some courses	Not have	107	1.50 ± 0.589	< 0.001
		Have	2	1.00 ± 0.00	
	Nationality	Iranian	112	1.46 ± 0.599	< 0.001
		Non- Iranian	2	2.00 ± 0.00	

*t-test

Further analysis revealed the positive correlations between the age and four questions including “Familiarity with the role of basic sciences knowledge in clinical settings” (r = 0.080), “Motivation toward learning more” (r = 0.047), “Familiarity with the roles and responsibilities of clinical students” (r = 0.015), and “Usefulness of participation in ground rounds” (r = 0.077). Three questions were presented negative correlation with the participants’ mean educational scores in questions such as “Familiarity with the role of basic sciences knowledge in clinical settings” (r= -0.020), “Familiarity with doctoring skills in medicine” (r= -0.004), and “Usefulness of participation in ground rounds” (r= -0.032). Moreover, there was only one positive correlation between “Familiarity with doctoring skills in medicine” and participants’ national university entrance exam rank (r = 0.037) (Table 3).

**Table 3:** Students’ views towards early clinical exposure and its correlation with their age, mean educational score, and university entrance exam rank

Question	Age	Grade point average	University entrance exam rank
**Familiarity with the role of basic sciences knowledge in clinical settings**	R^*^=0.80 P-value = 0.391	R^*^= -0.20 P-value = 0.863	R^*^= -0.062 P-value = 0.587
**Familiarity with doctoring skills in medicine**	R^*^= -0.033 P-value = 0.726	R^*^= -0.004 P-value = 0.975	R^*^= -0.037 P-value = 0.749
**Motivation toward learning more**	R^*^= 0.047 P-value = 0.616	R^*^= 0.118 P-value = 0.302	R^*^= -0.182 P-value = 0.109
**Familiarity with the roles and responsibilities of clinical students**	R^*^= 0.015 P-value = 0.873	R^*^= 0.174 P-value = 0.131	R^*^= -0.197 P-value = 0.086
**Usefulness of participation in ground rounds**	R^*^= 0.077 P-value = 0.406	R^*^= -0.032 P-value = 0.781	R^*^= -0.020 P-value = 0.862
**Providing opportunities to discuss and share knowledge**	R^*^= -0.052 P-value = 0.580	R^*^= 0.335 P-value = 0.003	R^*^= -0.159 P-value = 0.166
**Utility of early clinical exposure and providing more experiences**	R^*^= -0.060 P-value = 0.522	R^*^= 0.111 P-value = 0.331	R^*^= -0.206 P-value = 0.068

*Pearson correlation

### Qualitative Assessment

Overall, among a total of 118 participants in the study, 46 (38.98%) completed at least one of the qualitative questions. According to the students’ reports, the major strengths of the program were familiarizing with the clinical fields, having excellent instructors, performing excellent training, and teaching physical examinations very well. On the other hand, the major weakness of the program was due to its short duration and the high population of participants in each group. Furthermore, the most prevalent (n = 8, 17.39%) suggestion of the participants was to reduce the population of the students in each group for future programs. Table 4 shows the qualitative views of the students toward early clinical exposure in detail.

**Table 4:** Qualitative assessment of the students’ views towards early clinical exposure and their major comments about the program

Participants’ overall comment	Frequency of responses [n/all responses of the field] (%)	Examples of the participants’ statements
*Strength points of the program*		
**Having a clear goal**	3/42 (7.14%)	• “This course leads to decrease my confusion toward hospital and its clinical wards” • “The present course has a clear goals which made me interested in it”
**Respectful behaviors of the instructors**	4/42 (9.52%)	• “All the instructors were behaved to the participants and patients with full respect”
**Performing excellent training**	8/42 (19.04%)	• “The instructors were teaching the topics in an excellent form with patience”
**Teaching physical examinations very well**	13/42 (30.95%)	• “The instructors were completely dominant in the physical examination and how to teach it”
**Intimate atmosphere**	3/42 (7.14%)	• “The course atmosphere was so intimate which make us more interested in the program”
**Well planned**	1/42 (2.38%)	• “The course plan was so interesting and regular”
**Excellent instructors**	6/42 (14.28%)	• “The course instructors not only were completely dominant to the topics but also teach them very well”
**Teaching the communication skills and how to behave with the patients**	6/42 (14.28%)	• “The course made me familiar with the professional behavior and dressing with the patients” • “It was so interesting that the instructors give the chance to the students to perform physical examinations on their own” • “The course teaches us how to have an effective communication with patients”
**Problem-based teaching**	1/42 (2.38%)	• “The present course make a new insight for me into the problem-based learning and teaching in clinical challenges”
**Familiarity with the clinical field**	21/42 (50%)	• “This course makes me familiar with clinical wards which lead to decrease my fear about the hospital” • “This program not only familiarized me with the hospital but also reduced my fear of contacting patients” • “The present program makes me familiar with the hospital wards and how to fill out the patients’ medical records” • “It was interesting to contact with real hospital atmosphere which was not experienced in the routine educational curriculum”
*Weakness points of the program*		
**Short duration of the course**	12/22 (54.54%)	• “The course duration was too short” • “The program duration was not enough for all of the taught topics and it was better to expand the program time” • “It was better to allocate more time to the history taking and physical examination”
**High population of the participants in each group**	7/22 (38.81%)	• “The groups were so populated, it is suggested to divide participants into less populated groups” • “High density of the population in each group lead to having less time for clinical practice”
**Inadequate coordination**	5/22 (22.72%)	• “The executive team was not coordinated properly”
**Poor announcements about the program**	2/22 (9.09%)	• “The announcements of the program were not for all of the students”
**Course compactness**	1/22 (4.54%)	• “The course was too compacted, it was better to teach physical examination in two separate sections”
**Hospital crowdicity**	1/22 (4.54%)	• “The hospital was too crowded which reduced my learning ability”
*Participants’ suggestions toward the program*		
Increase the number of the practical classes	8/18 (44.44%)	• “ It was better to teach the practical topics in two or more sessions except in only one session”
Reduce the number of participants in each group	8/18 (44.44%)	• “The groups were too crowded which lead to reduce the participants ability to learn and practice the topics, please reduce the number of participants in each group for further programs”
Held theoretical classes in person	1/18 (5.55%)	• “Please hold both theoretical and practical sessions in person”
Make a chance for all participants to examine patients	1/18 (5.55%)	• “As the groups were too crowded it was hard for everyone to perform physical examinations, it is suggested to modify the number of the participants of each group for the future courses”

## Discussion

The present study, which evaluated the attitude of the undergraduate medical students of Mashhad University of Medical Sciences, Mashhad, Iran who are transferring from the pre-clinical course to the externship course, revealed that utilizing the ECE program in the period between the pre-clinical course and the externship course was almost interesting and valuable for the high rate of the participants who had a satisfied overview toward the program. In fact, the present ECE program generally leads to high “familiarity with the role of basic sciences knowledge in clinical settings”, “familiarity with doctoring skills in medicine”, “motivation toward learning more”, “familiarity with the roles and responsibilities of clinical students”, “usefulness of participation in ground rounds”, “providing opportunities to discuss and share knowledge”, and “utility of early clinical exposure and providing more experiences”. These findings push us to the fact that the integration of basic science education with clinical training at an early stage makes learning more relevant for students giving them an insight into the psychological aspects of different diseases and increasing students’ self-awareness and satisfaction with their curriculum. Early exposure to the clinical environment can also motivate students and reduce the stress of meeting patients and increases their sense of empathy towards patients, as mentioned by Dornan et al. in a previous study [18].

Oppose to our expectation, we did not observe significant differences in most of the fields of the survey; however, non-Iranian participants’ attitude toward the ECE program was significantly different in filed which related to the “familiarity with doctoring skills in medicine”, “familiarity with the roles and responsibilities of clinical students”, and “utility of early clinical exposure and providing more experiences”; these findings reveal that the present program was more interesting for the international medical students who study in Iran which can be an attractive point to eager international students to a medical university. While studying, international students face various educational problems that make it difficult for them to continue their studies in the host country. The educational problems of international students may be related to weakness in understanding the Persian language, different educational systems, and inappropriate teaching methods. Considering linguistic problems, the results of two different studies by Nicholson and Briguglio showed that the greatest weakness of these students is in expressing questions and presenting personal views in class [19–21]. Another problem for international students in medical universities seems to be the different educational system; Jaffer and Colvin reported in their study that most international students who travel to another country to study find it difficult to adapt to such an unfamiliar education system [22]. Beyond the linguistic difficulties of the international students, it seems that the ECE program can cover the gaps in the educational system variety between the Iranian medical education system and other countries.

Moreover, in two items including “usefulness of participation in ground rounds” and “utility of early clinical exposure and providing more experiences” those who had a history of failing some courses had a significantly lower score than those who did not. It indicates that maybe to precept ECE as a useful educational method, students should first have strong foundations in basic sciences. Although there are new ways and trends to learn medical science in the pre-clinical period it’s still an inseparable part of medical education [18–20, 23]. Similarly, Duque et al. reported that medical students who had early clinical experience were usually more satisfied with their medical education [24]; Sathishkumar et al. reported that early exposure in endocrine physiology course in the first year of medical education was a valuable event, and medical students clearly enjoyed this experience [25].

Furthermore, there were positive correlations between age and some questions including “awareness of responsibilities of clinical students”, “motivation toward learning more”, and “knowing the role of basic sciences knowledge in clinical settings”. On the other hand, the students with the higher grade point average indicated a positive correlation with the following fields including “motivation toward learning more”, “familiarity with the roles and responsibilities of clinical students”, “providing opportunities to discuss and share knowledge’, and “utility of early clinical exposure and providing more experiences”; These findings suggest that the top participants were not completely revealed positive correlation with the program. While in Jafarzadeh et al study. Despite there not found exact similar previous studies to define the similar correlations, based on our search; however, Rawekar et al observed that medical students in higher courses and stages have more positive attitudes toward ECE [12]. Also, Jafarzadeh et al demonstrated variable findings to describe the correlation between age and efficacy of the ECE, while in some topics there were a significant correlation (e.g. remembering taught courses), and in some others were not (e.g. visualization of taught courses, increased interest, better understanding of taught courses, and improvement of learning process) [26]. Moreover, although ECE improved the academic performance of medical students in previous studies, we did not find an exact similar study that investigated the impact of students’ grades on their attitude toward ECE. These varieties in the finds between different studies could be due to two major reasons, including differences in their ECE interventional program and their scale to measure the impact of the intervention.

Participants have emphasized on strengths of this course, such as familiarizing them with clinical fields and providing excellent clinical skill and physical examination training by the professional trainers. In previous studies, different views have been expressed about learning practical methods and skills in ECE, some have declared its importance, while others have barely mentioned it [27–29]. But it is noteworthy that its importance has been expressed more significantly in longer courses rather than short courses like ours. Study participants also believed that the course was very intensive and short and it is better to extend the duration of the course to have more long-term effects on attitudes and behavior. However, the positive effects of this short course still can be good evidence to conduct more regular and longer courses in the future. It was mentioned that the frequency of students in each group was too many and without any doubt, it had negative effects on educational outcomes.

Despite the strong points of our study which included consisting of a large frequency of undergraduate medical students, defined a unique ECE program for undergraduate medical students, and the program conducted during the Coronavirus Disease 2019 (COVID-19) pandemic when the virtual education made too harmful effects on the medical students’ learning [30, 31]; there were some major limitations including the compact duration of the program, the few international participated medical students, and the high population of the participants in each group.

## Conclusions

Providing early clinical exposure can enhance students’ motivation and understanding of the role they will play in the future as a physician. The ECE program was shown to have the potential benefits to guide the policymakers and other stakeholders to provide a framework for the integration of basic and clinical sciences. Moreover, due to the positive effect of the ECE program, it is suggested to implement it as a part of the medical education curriculum in medical universities; however, further studies are required to evaluate the long-term effects of the ECE program, find out its effects on the physician’s future clinical work, and modify the program content.

## Data Availability

The data that support the findings of this study are available on request from the corresponding author. The data are not publicly available due to privacy or ethical restrictions.

## List of Abbreviations

ECE: Early Clinical Exposure
HIS: Hospital Health Data System
PACS: Picture Archiving and Communication System
SPSS: Statistical Package for the Social Sciences
COVID-19: Coronavirus Disease 2019
